# Sputum handling for rheology

**DOI:** 10.1038/s41598-023-34043-9

**Published:** 2023-05-11

**Authors:** Lydia Esteban Enjuto, Matthieu Robert de Saint Vincent, Max Maurin, Bruno Degano, Hugues Bodiguel

**Affiliations:** 1 Univ. Grenoble Alpes, CNRS, Grenoble-INP, LRP UMR5520, Grenoble, France; 2 Rheonova, 1 Allée de Certèze, 38610 Gières, France; 3 Univ. Grenoble Alpes, CNRS, CHU Grenoble Alpes, TIMC, Grenoble, France; 4 Univ. Grenoble Alpes, INSERM U1030, CHU Grenoble Alpes, Grenoble, France

**Keywords:** Biophysics, Biotechnology, Biomarkers, Diseases, Medical research, Materials science, Physics

## Abstract

The rheology of sputum is viewed as a powerful emerging biophysical marker for monitoring muco-obstructive pulmonary diseases such as cystic fibrosis (CF) and non-CF bronchiectasis (NCFB). However, there is no unified practice to process sputa from collection to analysis, which can lead to highly variable, and sometimes inconsistent results. The main objective of this study is to bring light into the handling of sputum samples to establish a standardised and robust protocol before rheological measurements. Sputum collected from 22 CF and 10 NCFB adults, was divided into control (vortexed and fresh: non-heated and non-frozen) and three treated conditions (either non-vortexed, heated or frozen). In addition, 6 CF expectorations were used to study the dynamics of ageing over 24 h. Sputum’s mechanical properties were measured with a rotational rheometer to obtain their properties at rest, elastic ($G'$) and viscous moduli ($G''$), and at the onset of flow, critical deformation ($\gamma _c$) and critical stress ($\sigma _c$). We demonstrate that heating sputum is completely destructive while freezing sputa at $-80\,\,^{\circ }\hbox {C}$ has no discernible effect on their rheology. We also show that the variability of rheological measurements largely resulted from the sample’s macroscopic heterogeneity, and can be greatly reduced by non-destructive vortex homogenisation. Finally, we observed contrasted ageing effects as a fonction of purulence: while the viscoelasticity of purulent samples reduced by half within 6 h after collection, semi-purulent samples did not evolve. These results guide towards a robust unified protocol for simple sputum handling in rheometry. We therefore suggest to vortex and snap freeze sputum samples immediately after collection when direct testing is not possible.

## Introduction

Muco-obstructive lung diseases as defined by Boucher^1^ clinically present “cough, sputum production, and episodic exacerbations” leading to the obstruction of the airways. These diseases include cystic fibrosis (CF), non cystic fibrosis bronchiectasis (NCFB), primary ciliary dyskinesia (PCD) and chronic obstructive pulmonary disease (COPD).

In these diseases, bronchial mucus is hyper-secreted and thickened, which results in anomalous mucus clearance, thereby favouring bacterial growth and subsequently infection^2,3^. Techniques to help patients drain the mucus out of their airway system involve chest physiotherapy^4^, and the use of mucolytic treatments which directly alter the mucus structure^5,6^. These methods consist in either altering the breathing airflow to improve mucus mobilisation, or reducing mucus viscoelasticity, two physical concepts which can be quantified through rheology. The elastic ($G'$) and viscous moduli ($G''$) assess the viscoelastic character of mucus at rest, and the critical stress ($\sigma _c$) and deformation ($\gamma _c$) determine its flowing ability.

The potential of sputum rheology as a direct biomarker of health status has been brought forward since the 1980s^7–11^. Measuring the rheological properties of sputum indeed presents two decisive advantages. First, mucus is readily available from obstructive pulmonary disease patients with airway obstruction in the form of sputum. Sputum is expelled through the mouth, during a physiotherapy session for instance, by NCFB or CF patients who have not been treated with a CFTR regulator. Second, rheology quantitatively characterises physical properties linked to mucus transport. Despite this interest, sputum rheology has not yet been adopted in routine as the quantitative link between rheology and bronchial obstruction still remains an open question^12–14^. One of the present limitations resides in the fact that there is no standardised protocol on the way samples should be treated prior to measurement. Moreover, the immediate access to a rheometer, as well as to an expert choice of measurement protocols, is not always possible for many clinical research teams. Having a consistent procedure to store samples with no effect on rheology would encourage multidisciplinary collaborations making rheology more accessible.

The development of sputum rheology also poses several practical questions. The first one relates to the rheological measurement itself, in terms of spatial as well as temporal scale to be investigated. Micrometre-scale rheology techniques, also referred to as microrheology^15–17^, are indeed relevant to probe the mucus environment as it would be perceived at the scale of cilia, and thereby assess the effectiveness of mucociliary clearance^14^. Although these techniques do not require large volumes of sample, they imply seeding (sub-)micrometric probe particles therein, and often require specific instrumentation and competency. Macrorheology at the other end considers the mucus sample as a whole, which may be more representative of its global transport properties. Rheological measurements typically consist in exerting oscillatory strains (or stresses) and measuring the resulting stresses (or strains) through the sample. Oscillatory sets with gradually varying frequency probes the structure of the sample at different time scales. In bronchial mucus, the elastic and viscous moduli typically follow weak power laws with frequency in the range 0.1–10 Hz with $G' > G''$^17–20^, which illustrates a soft gel-dominated behaviour from the time scale of the ciliary beating motion to that of mucus drainage. Frequency tests do not give any information on the behaviour of mucus under large deformation. Conversely, increasing the amplitude of oscillations, at fixed frequency, allows to probe the mucus behaviour from quasi-rest up to flowing conditions^18,20^, and therefore simulate more representatively the wide range of strains that can be exerted through the various natural and assisted clearance mechanisms.

Due to its relative simplicity of operation, macrorheology is often preferred in preclinical studies. Visual inspection of CF sputa evidences that they are in general composed of two phases: a light and clear matrix, and thick, opaque mucous plugs dispersed therein. This heterogeneity is problematic in rheometry as the samples tested may over-represent one of these two rheologically distinct phases. A proposed solution consists in homogenising samples prior to measurements by vortexing, but it is still debated as potentially disruptive to the sample’s structure^9,10,21^. Other groups prefer to isolate the plugs before analysis^22^, through a protocol that complicates the direct use of sputum rheology in the hospital’s routine.

Another immediate question, particularly acute in the context of the Covid-19 pandemic, is linked to biosafety when manipulating potentially contaminated samples. In the field of tuberculosis, Friedrich et al.^23^ had already proved the inactivation of *Mycobacterium tuberculosis* by heating sputum. This inactivation could eliminate the need for category 3 BSL (biosafety level). The same way, in the context of SARS-CoV-2, preventive heating of samples to inactivate the SARS-CoV-2 virus has been suggested^24,25^, allowing immediate analysis of sputum without waiting for its detection through q-PCR. The effect of heating on the structure of sputa is however unknown. Alternatively, delaying the measurement raises the question of sample long-term conservation. The freezing of sputa has been validated for cell count after storage at $-20\,\,\,^{\circ }\hbox {C}$ with the use of cryopreservants^26^, and for bacterial DNA detection at $-80\,\,\,^{\circ }\hbox {C}$^27^. The few existing rheological tests on thawed sputa have been carried out after freezing at $-20\,\,\,^{\circ }\hbox {C}$ without additives^28^, or at $-80\,\,\,^{\circ }\hbox {C}$ mixing results of expectorations coming from different obstructive diseases^29^. Without freezing, samples can rapidly undergo alterations, including enzymatic degradation^30^, alteration of bacterial components^28,31^ and oxidation of mucins^32^. Thus, the effect of ageing on rheology must also be addressed as samples cannot always be treated rapidly.

In the present paper, we address these questions from a practical standpoint, to progress towards a unified protocol for sputum rheology in CF and NCFB. Starting from fresh, vortex-homogenised samples as reference, we first quantify the factors of variability within the sputum sample population and within aliquots. We then systematically assess the effect of non-homogenisation, heating, direct freezing and snap freezing by cross-comparing ‘treated’ (prepared with the condition to be assessed) to ‘control’ (prepared with the reference protocol) aliquots of each sample. This direct cross-comparison helps us to overcome the limitation of sample variability. Finally, we address the effect of sample ageing through continuous rheological measurements under controlled atmosphere, evidencing a possible dichotomy according to the initial aspect of the samples. Our results provide guidelines towards protocol recommendations to perform sputum rheological measurements in routine.

## Methods

All experiments were carried out in accordance with relevant guidelines and regulations.

### Sputum processing

#### Clinical protocol

Sputum samples were obtained from the “Centre de Ressource et de Compétence de Mucoviscidose” at the Grenoble Alpes University Hospital. All patients included gave their written informed consent and their clinical information were anonymised and kept confidential. The study was approved by the French research ethics committee (Comité de Protection des Personnes, case number 20.09.08.61213).

#### Sample collection

Expectorations were induced by autogenic drainage^33^ with the help of a physiotherapist and immediately transferred into sterile flat bottom tubes at $4\,\,^{\circ }\hbox {C}$. Samples were macroscopically heterogeneous, composed of saliva, coloured bronchial mucus plugs and translucent mucus. The volume collected varied between patients and diseases. NCFB expectorations were larger than CF samples with volumes ranging from 0.55 to 7 ml while CF samples were between 0.4 and 5 ml. Purulence was determined by visual inspection following the colour classification proposed in ref.^2^. Expectorations colours ranged from clear to dark yellow/green. A small fraction (0.2 ml) was used to test the presence of SARS-CoV-2 by reverse-PCR. All samples of the study tested negative for this virus. Then, within 1 h after sample collection, saliva was removed and, unless otherwise stated, the expectoration was homogenised. The sample was vortexed, gently increasing the stirring intensity until the sample visually takes the shape of a torus and starts flowing (see Supplementary Fig. S1 for specific details). This vortex intensity was then maintained during 30 s. Visually, all aliquots looked homogeneous after this procedure. We decided to use the vortexed samples as controls, since, as will be shown in the results, variability was greatly increased for the non-vortexed ones, in particular for the case of CF. When possible, samples were then divided into at least two aliquots. Each sample thus provided at least one aliquot for the studied condition, and at least one control, so that treated and control samples could be cross-compared. Whenever additional aliquots were obtained, they were used as replicates. The typical volume per aliquot was about 500 $\upmu$l, which allowed reliable rheological measurements (330 $\upmu$l required).

#### Treatments

We investigated the effect of four different conditions by comparison with controls (fresh vortexed samples):

- Heating ($N_{\textrm{CF}} = 3$)—fresh samples were homogenised and heated at $56\,\,\,^{\circ }\hbox {C}$ for 30 min^24,25^. The samples were then let 15 min to cool back to room temperature.
- Freezing ($N_{\textrm{CF}} = 4$)—fresh samples were homogenised and brought into a $-80\,\,\,^{\circ }\hbox {C}$ freezer. Prior to measurement, they were thawed quickly at $37\,\,^{\circ }\hbox {C}$.
- Snap freezing ($N_{\textrm{CF}} = 7$, $N_{\textrm{NCFB}} = 4$)—fresh samples were homogenised and put into dry ice for rapid freezing before storage in the $-80\,\,^{\circ }\hbox {C}$ freezer. Prior to measurements, they were thawed quickly at $37\,\,^{\circ }\hbox {C}$.
- Not vortexing ($N_{\textrm{CF}} = 8$, $N_{\textrm{NCFB}} = 4$)—fresh samples were not homogenised prior to sample aliquoting. Visually, non-homogenised CF aliquots featured large (several mm) thick plugs within a slightly turbid liquid matrix; instead, non-homogenised NCFB samples looked monophasic.

In addition, we also assessed the ageing of $N_{\textrm{CF}} = 6$ samples when kept at room temperature during 24 h.

#### Data analysis

Statistical analysis was performed with Matlab R2020b. We verified normality with the Shapiro-Wilk test and compared control from treated samples by bilateral t-tests.

### Rheology

#### Strain sweeps

Rheological measurements were performed with a rotating rheometer, Rheomuco (Rheonova, France), operating in oscillation at 1 Hz and $37\,\,^{\circ }\hbox {C}$, with rough, plane-plane geometries (25 mm diameter). The gap between the plates was dependent of the volume of the aliquot and ranged from 0.5 to 1 mm. Samples were submitted to strain steps of increasing amplitude between 0.1 % and 10,000 %; due to low signal-to-noise ratio the measurements obtained below 1 % strain were systematically ignored. The values of the elastic and viscous moduli were automatically extracted at 5 % strain ($G'_{5\%}$ and $G''_{5\%}$), as well as the critical strain $\gamma _c$ (where $G' = G'' = G'_c$) and the corresponding critical stress $\sigma _c = \sqrt{2} G'_c \gamma _c$. As these rheological quantities follow log-normal distributions^20^, we define $g' = \log _{10} G'$, $g'' = \log _{10} G''$, $d_c = \log _{10} \gamma _c$, and $s_c = \log _{10} \sigma _c$, with $G'$, $G''$ and $\sigma _c$ expressed in Pascals, and $\gamma _c$ unit less.

#### Ageing of sputum

Ageing experiments consisted in continuously exerting a low-amplitude (5 %) oscillating strain during 24 h with an ARG2 rheometer (TA Instruments). The sample was kept at room temperature ($25\,\,^{\circ }\hbox {C}$) within an isolation chamber, under a constant flow of water-saturated air to preserve it from drying.

## Results

In this study 26 expectorations were collected from 22 stable CF patients, 13 men and 9 women with ages from 19 to 53 years (33.3 ± 9.6), and 10 expectorations from NCFB patients, all women between 19 and 45 years (31.4 ± 8.2). Demographic and other relevant patients data are summarised in the Supplementary Table S1.

**Figure 1:**
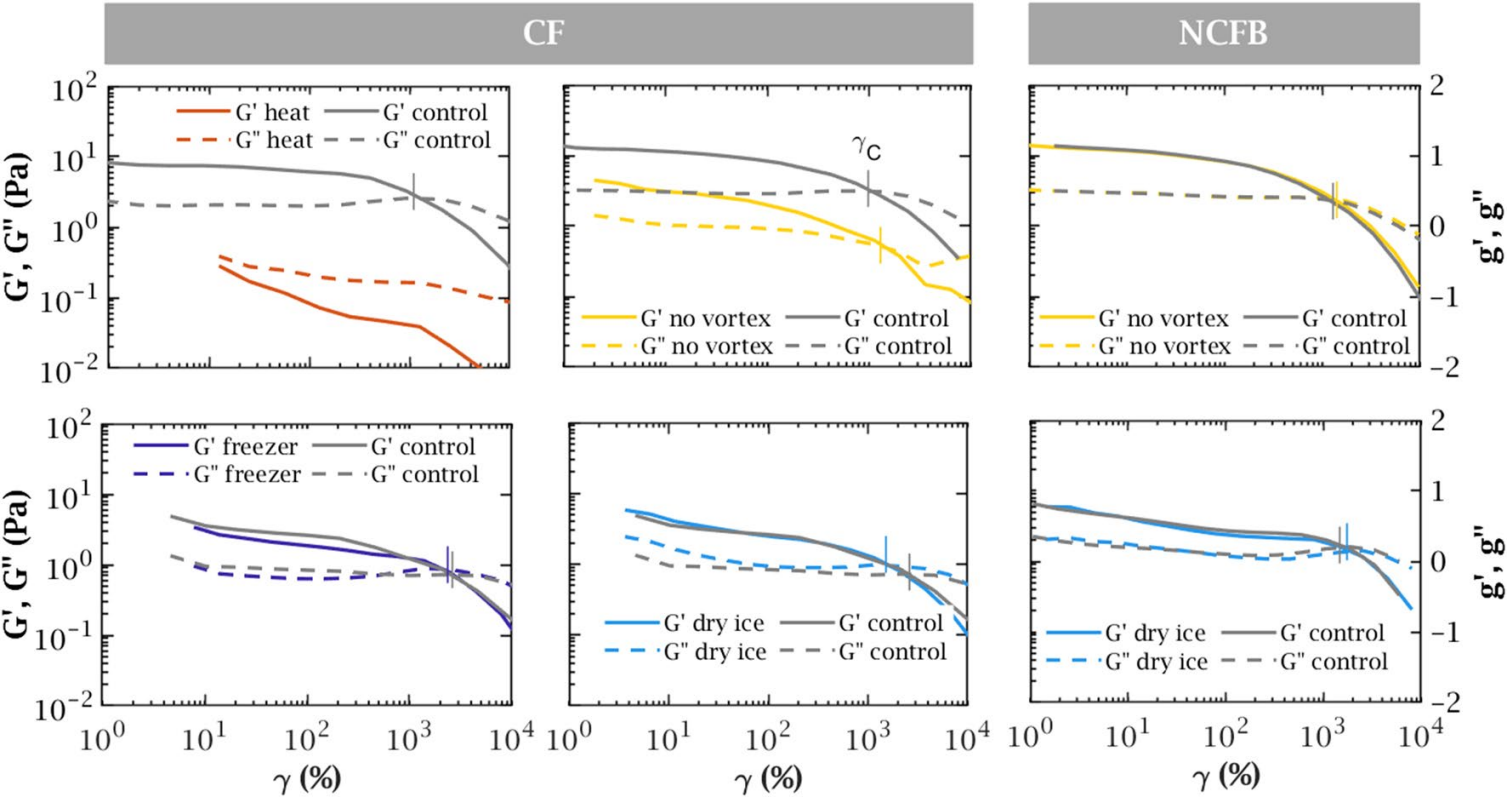
Representative strain sweeps for CF and NCFB sputa: evolution of $G'$ (solid line) and $G''$ (dashed line) with the exerted strain. Grey and coloured curves correspond to control and treated samples, respectively. The mark at the crossing of the curves denotes the critical point, where $G' = G''$ at $\gamma = \gamma _c$. Measurement uncertainty lies within the line thickness.

### Rheological parameters

#### CF and NCFB sputa are rheologically similar

Rheologically, sputum behaves like a soft gel with very high stretching ability, partly returning to its original state when released and thereby showing a predominant elastic response. Under strong efforts, beyond a critical deformation $\gamma _c$ or stress $\sigma _c$, some bonds in the network break. The gel then looses most of its elastic character and flows. Figure 1 illustrates this general behaviour in CF and NCFB sputa. Under low strain, sputa feature a constant linear viscoelastic regime (plateau in $G'$ and $G''$) with $G' > G''$, for strains up to several tens of percent. Increasing the strain, $G'$ and $G''$ gradually drop until intersecting at the critical strain $\gamma _c$, typically around 1000%.

**Figure 2:**
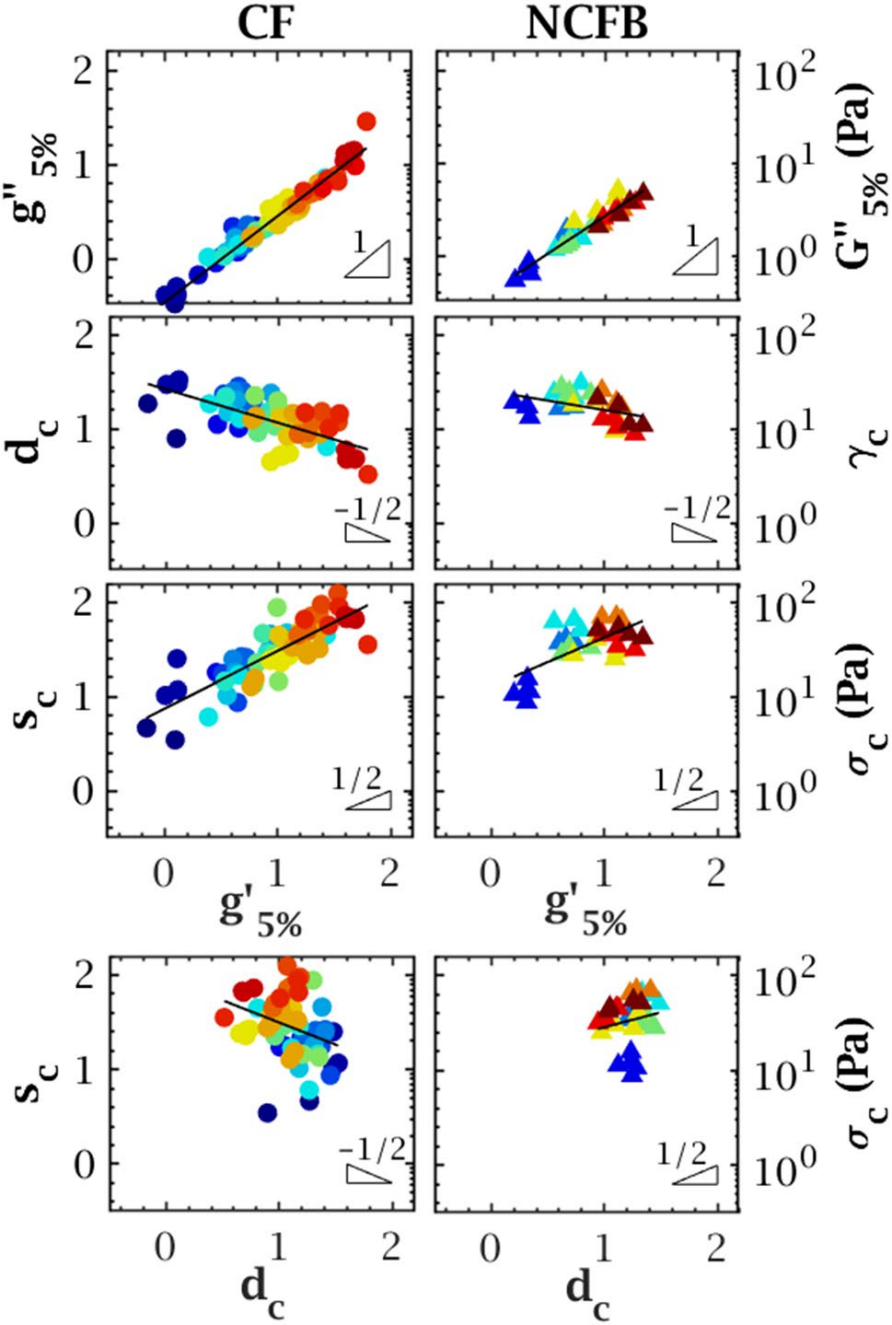
Top: Evolution of the logarithmic viscous modulus $g''_{5\%}$, critical strain $d_c$ and critical stress $s_c$ with the logarithmic elastic modulus $g'_{5\%}$ for non-heated expectorations of CF ($N=26$) and NCFB ($N=10$) patients. Each colour represents an expectoration with its corresponding aliquots. The uncertainty on the moduli at 5 % strain is in the order of 0.6 Pa, smaller than the symbol size through most of the distribution. $g'_{5\%}$ and $g''_{5\%}$ are strongly correlated, and $d_c$ and $s_c$ are weakly correlated to $g'_{5\%}$. Bottom: Evolution of $s_c$ with $d_c$. No correlation is found between these variables.

We summarise this general behaviour by four rheological quantities: the elastic and viscous moduli in the linear regime, $G'_{5\%}$ and $G''_{5\%}$, and the critical strain and stress, $\gamma _c$ and $\sigma _c$. In CF sputa, $G'_{5\%}$ and $G''_{5\%}$ typically range within 1–100 Pa and 0.3–30 Pa, respectively; $\gamma _c$ within 300–3000 % and $\sigma _c$ within 3–100 Pa, or equivalently $g'_{5\%} \in [0,2]$, $g''_{5\%} \in [-0.5,1.5]$, $d_c \in [0.5,1.5]$ and $s_c \in [0.5,2]$ in logarithmic scale (Fig. 2). In NCFB expectorations, $G'_{5\%}$ and $G''_{5\%}$ range within 1–20 Pa and 0.5–5 Pa, $\gamma _c$ within 900–3000 % and $\sigma _c$ within 7–70 Pa, or $g'_{5\%} \in [0,1.5]$, $g''_{5\%} \in [-0.5,1]$, $d_c \in [0.5,1.5]$ and $s_c \in [0.5,2]$. CF and NCFB sputa thus lie within the same magnitude range regarding these four rheological parameters.

#### Linear and flow rheology are poorly correlated

For both CF and NCFB expectorations, in the linear regime, the viscous and elastic moduli are highly correlated as the ratio $G''_{5\%}/G'_{5\%}$, referred to as the damping ratio $\tan \delta _{5\%}$, is consistently close to 0.3. The critical strain and stress are poorly correlated to $G'_{5\%}$ as the slopes are flatter and the measured values are highly dispersed (Fig. 2). In general, higher viscoelasticity comes with higher critical stress and lower critical strain. Lastly, we could not identify any correlation between $d_c$ and $s_c$. We therefore focus, in the present study, on three weakly correlated or independent variables: $g'_{5\%}$, $d_c$ and $s_c$.

### Sample and aliquoting variability

We have first divided 15 CF and 10 NCFB control samples into 2, 3 or 4 aliquots to assess sputum’s inter-sample (between expectorations) and intra-sample (between aliquots) variability.

#### Rheometric variables are log-normally distributed

**Figure 3:**
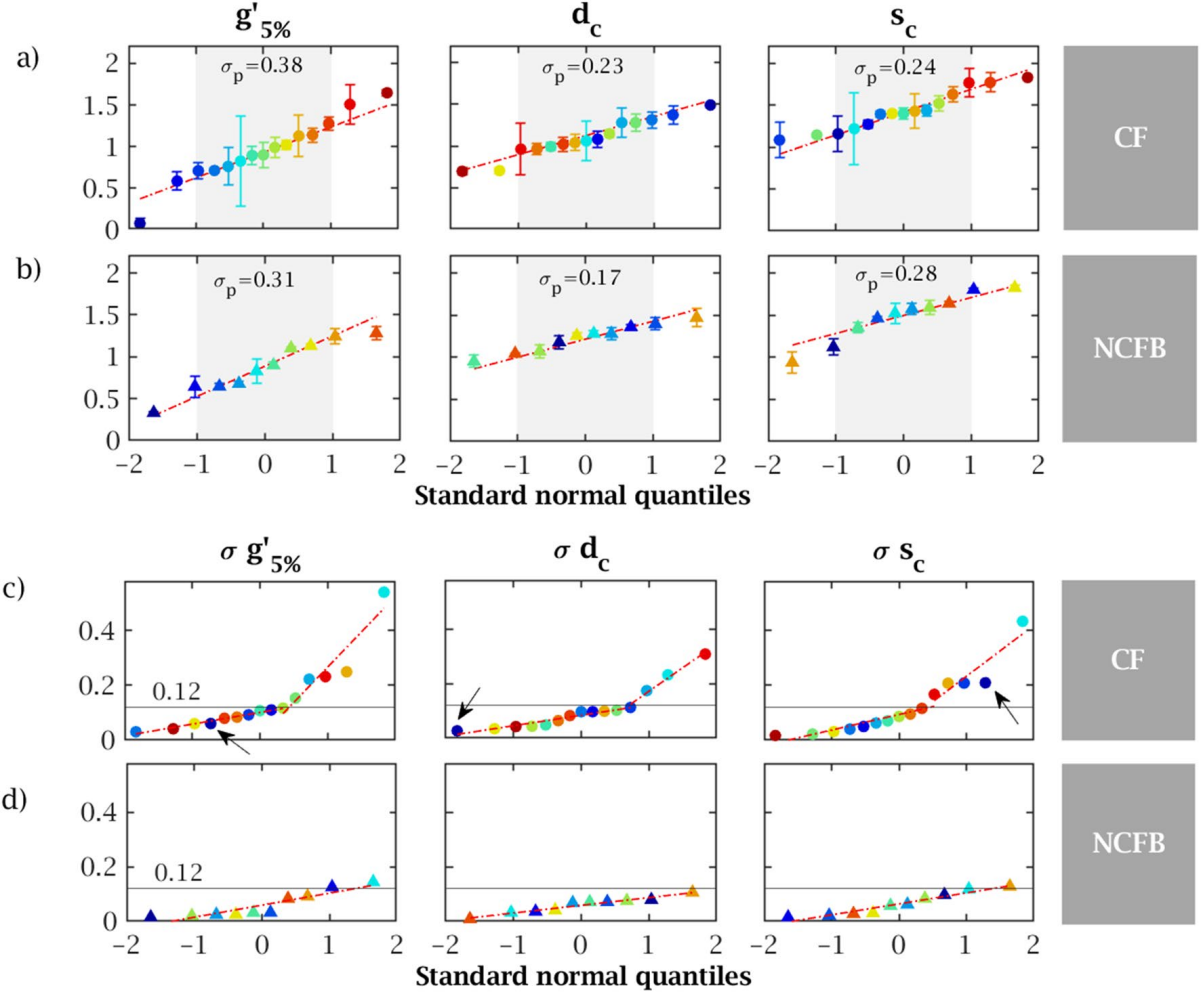
(**a**,**b**) Distribution of the logarithmic rheological quantities for the 15 CF (circles) and 10 NCFB (triangles) control expectorations. Each data point corresponds to the average value per sample, identified by its colour. The error bars correspond to the standard deviations due to sample aliquoting. The dashed line corresponds to a normal distribution; the grey area delimits 1 standard deviation around the mean. (**c**,**d**) Distribution of the standard deviations between the aliquots of the 15 CF (circles) and 10 NCFB (triangles) control expectorations.

Figure 3a,b depicts the distribution of the logarithmic values of the three quantities considered ($g'_{5\%}$, $d_c$ and $s_c$), represented as quantile-quantile plot with normal distribution. Each data point corresponds to the average value per sample, identified by its colour; the associated standard deviations between aliquots are represented as error bars. All data sets are very well adjusted by normal distributions in accordance with Patarin et al.^20^. Shapiro-Wilk normality tests provide p-values of respectively 0.82, 0.61 and 0.37 for $g'_{5\%}$, $d_c$ and $s_c$ for CF samples, and 0.63, 0.88 and 0.45 for NCFB samples. The normality is therefore verified. As a consequence, the effects of treatments were analyzed using log-transformed data. In particular, bilateral t-tests for mean equality check are made on these transformed data. Note that by doing so, the tests check the equality of the geometric means of the scaled variables and not that of the arithmetic mean^34^, the latter being often biased by large outliers.

#### Rheometry is reproducible within aliquots

At the individual sample level, the standard deviation between aliquots quantifies the measurement reproducibility. Throughout the CF population, the distribution of standard deviations took a piecewise linear form, which corresponds to a bimodal probability distribution (Fig. 3c). In the low-variability subpopulation, which encompasses 65–70 % of the samples, the aliquoting standard deviation remained below 0.12, between half and third of the inter-sample variability $\sigma _p$ obtained in Fig. 3a,b. In contrast, the distribution of standard deviations for NCFB samples (Fig. 3d) followed a normal distribution (linear quantile plot) and all values stayed below 0.12. This lower variability in NCFB samples is consistent with their visually lower heterogeneity (Supplementary Fig. S2).

Another remarkable feature is that the variability in each of the rheological variables is quite independent. For instance, the CF sample marked with an arrow in Fig. 3c featured a high $s_c$ standard deviation, while the standard deviations regarding the other two variables are small.

### Treatment effect

**Figure 4:**
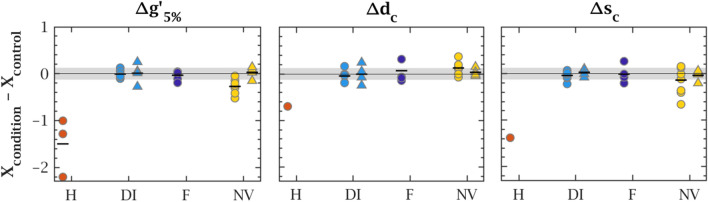
Comparison between the logarithmic rheological quantities of treated and control. samples. H: heating, DI: Dry Ice, F: Freezing at $-80\,\,^{\circ }\hbox {C}$ and NV: not vortexed. Symbols correspond to individual CF (circles) and NCFB (triangles) samples. Only one of the three heated samples featured a crossover point, resulting in a single point in $\Delta d_c$ and $\Delta s_c$ for this condition. The small horizontal bar denotes the mean value of the considered condition. The grey band stands for $\pm 0.12$, the aliquoting standard deviation which separates low from high variability CF samples.

We first analysed the effect of the treatments by qualitative inspection of the rheological curves of control and treated aliquots from the same sample (Fig. 1). Then, we compared sample by sample the treated and control aliquots by calculating the difference between the corresponding logarithmic rheological values, $\Delta X = X_{\textrm{condition}} - X_{\textrm{control}}$, where $X = g'_{5\%}$, $d_c$ and $s_c$. The obtained values for CF and NCFB samples are shown in Fig. 4 as circles and triangles respectively. These results were statistically confirmed with bilateral t-tests to inspect mean equality between treated and control groups. The null hypothesis establishes mean equality between the groups while the alternative hypothesis considers mean inequality.

#### Heating degrades the structure of sputum

Heating sputum qualitatively modified its rheology (Fig. 1, top left). Samples became much more fluid-like ($G'' > G'$) throughout the whole strain range, with viscoelasticity levels decreased by more than one order of magnitude, and lost their linear viscoelastic plateau. Only one of the three samples tested featured a critical point, observed at both lower strain and stress than the corresponding control (Fig. 4). Structurally, this reversal from a mostly energy-conservative to a mostly energy-dissipative material means that the original backbone network of sputum has turned into non-cohesive elements, without recovering as the sample went cooled back to room temperature. These observations demonstrate the degradation of the gel network under heating.

#### Freezing has no effect on sputum rheology

Conversely, both CF and NCFB frozen samples were rheologically almost indistinguishable from their fresh counterparts (Fig. 1, bottom), although direct freezing caused slightly more variability than snap freezing in dry ice for the analysed CF samples (Fig. 4). P-values (Supplementary Fig. S3) for the freezing treatment were in all cases higher than 0.75, therefore the equality of means is very likely.

#### Vortex preserves rheology and reduces variability

Vortexing samples did not alter the qualitative shape of the rheological curves (Fig. 1, bottom right). However, non-vortexed CF samples featured weak variations of the mean: slightly lower $g'_{5\%}$ and $s_c$, and slightly higher $d_c$ (Fig. 4). Visual inspection of CF samples prior to measurement showed no difference in heterogeneity as shown in Supplementary Fig. S2. We believe the lower viscoelasticity observed in non-homogenised samples could be due to the fact that the highly viscoelastic plugs would rather stay in the centre of the rheometer’s geometry while the matrix flows towards the edge of the geometry. Since stresses at the outer edge of the plates have a dominant contribution to the overall torque generated, the measured response would then be mostly determined by the matrix, of comparatively low viscoelasticity. Conversely, vortex homogenisation breaks these inclusions and therefore allows to get a higher proportion of this viscoelastic material evenly distributed on the geometry, which could explain why homogenised samples are actually measured more viscoelastic. Statistically, we can reject the alternative hypothesis, different means (p > 0.05) for all the samples (Supplementary Fig. S3). However, the variation in rheological quantities was significantly higher than the aliquoting standard deviation after homogenisation (grey band in Fig. 4) for the CF samples. Therefore, the effect of non-vortexing is more important that the aliquoting effect. This difference thus highlights the importance of homogenisation to get meaningful results, especially in samples which present large and thick mucous heterogeneities. In contrast, vortex did not have any effect on NCFB expectorations and this result was consistent for all the analysed samples: all t-tests gave p-values over 0.75. This outcome was expected as, by sight, NCFB sputa are less heterogeneous than CF expectorations.

In summary, our results show no effect of freezing on sputum rheology, while heating is completely destructive. They also confirm the importance of carefully homogenising CF samples as their heterogeneity is a major factor of measurement variability. The specific effect of the vortexing protocol (e.g. duration, intensity) on this variability, especially in the samples with important aliquoting standard deviation in Fig. 3b), remains an open question.

### Ageing of sputum

Biological samples evolve and should therefore be analysed immediately after collection. However, this temporal evolution is rarely quantified and it is therefore difficult to assess the sensitivity of samples to ageing. We characterised the rheological evolution of CF sputa when left at room temperature during 24 h by continuously monitoring their linear elastic and viscous moduli. Measurements started 1 h after collection.

**Figure 5:**
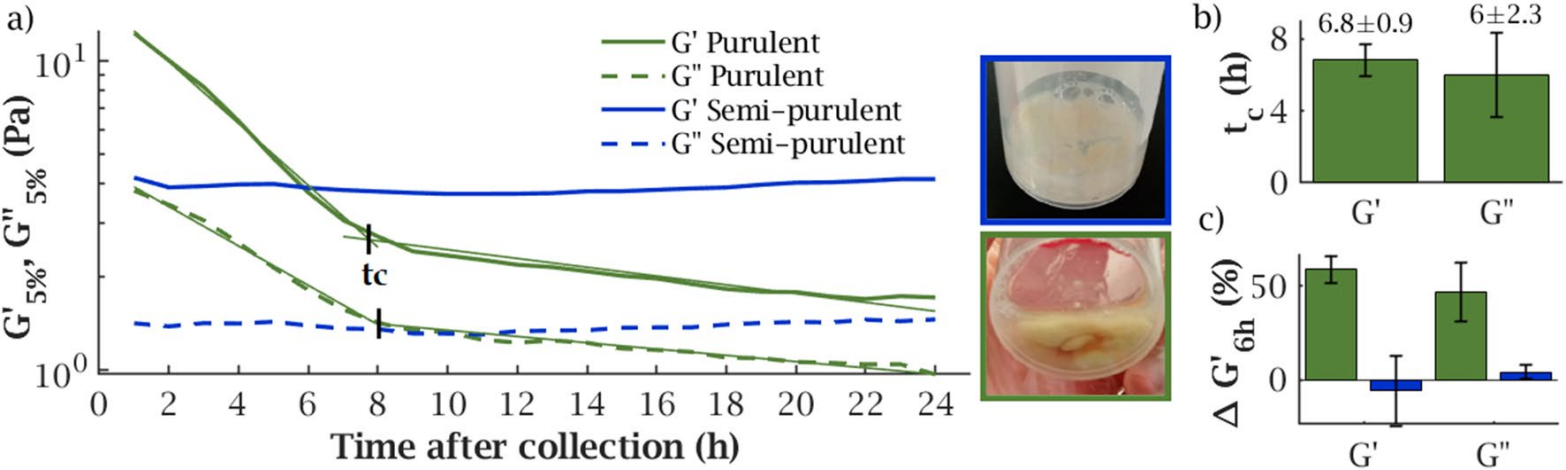
(**a**) Storage and loss moduli evolution in 24 h for a purulent (green) and a semi-purulent samples (blue). The crossing point of the fits gives the characteristic time $t_c$ (indicated with black marks) for the $G'$ and $G''$ drop found in purulent samples, obtained by intersecting the exponential fits to the two regimes (light green lines). (**b**) Mean values and standard deviations of $t_c$ in $G'$ and $G''$ for purulent samples. (**c**) Mean values and standard deviations of the evolution of $G'$ and $G''$ after 6 h, $\Delta G'_{6h}$ and $\Delta G''_{6h}$ for purulent and semi-purulent samples.

#### Semi-purulent sputa are rheologically stable

Sputa featured two distinct evolutions according to their purulence, estimated visually^2^ (Fig. 5). The semi-purulent samples ($N = 2$) did not show any substantial rheological evolution as the values of $G'_{5\%}$ and $G''_{5\%}$ remained constant throughout the whole test.

#### Purulent sputa degrade in 6 h

Conversely, in the purulent sputa ($N = 4$) $G'_{5\%}$ and $G''_{5\%}$ first dropped before an apparent stabilisation. The characteristic time to reach this steady state, $t_c$, was obtained by interpolation of the crossing point of two exponential fits in the two regimes found. It was very similar among the four samples, $t_c = 6.8 \pm 0.9$ h for $G'$ and $6.0 \pm 2.3$ h for $G''$ (Fig. 5b). Moreover, the initial dynamics was also remarkably consistent among purulent samples. The reduction in moduli after 6 h, defined as

$$\begin{aligned} \Delta G_{6\textrm{h}} = \frac{\Delta G_{5\%}(t=1\textrm{h}) - \Delta G_{5\%}(t=6\textrm{h})}{\Delta G_{5\%}(t=1\textrm{h})}, \end{aligned}$$

was $\Delta G'_{6\textrm{h}} = 59 \pm 5.7~\%$ and $\Delta G''_{6\textrm{h}} = 47 \pm 16~\%$. The difference between these two drops was not significant (p = 0.227). Semi-purulent samples did not evolve significantly as $\Delta G'_{6\textrm{h}} = -5.7 \pm 18.4~\%$ and $\Delta G''_{6\textrm{h}} = 4.2 \pm 3.6~\%$ (Fig. 5c).

A comparable decrease in CF sputum viscoelasticity, attributed to biological activity, was already reported at $37\,\,^{\circ }\hbox {C}$, but not at $25\,\,^{\circ }\hbox {C}$^28^. Conversely, Yuan *et al.* reported a two-fold increase of the viscoelastic moduli of healthy sputa under pure oxygen at $37\,\,^{\circ }\hbox {C}$, attributed to an increase of mucin cross-links due to oxidation^32^. Our own results suggest that oxidation actually plays a minor role in ambient atmosphere as no stiffening is observed. The distinct evolution of the purulent samples tends to confirm that proteolytic degradation of the sample has a greater effect than oxidation as previously reported for acute asthma samples^30^. Practically, this evolution confirms that the delay between collection and rheometry should be minimised. However, semi-purulent samples are much less sensitive and a delayed rheological characterisation could still be considered meaningful.

## Discussion

In this paper, we have systematically investigated the effect on rheology of sample handling and storage of cystic fibrosis and non cystic fibrosis bronchiectasis sputa.

We first demonstrated that both CF and NCFB expectorations can be stored at $-80\,\,^{\circ }\hbox {C}$ without affecting substantially their rheological properties. Therefore, the rheological analysis of sputum samples can be performed without complicated logistic procedures. For instance, heating for preventive decontamination of the samples is not necessary as the possibility of freezing allows to wait for the results of microbial analyses prior to handle them.

While sputum samples are easy to obtain, their heterogeneity poses a concern about the repeatability of macrorheological measurements. We have shown how vortexing CF and NCFB sputum reduces the dispersion of rheological parameters, while keeping the mean values unchanged. The measurements carried out with NCFB samples, which are more homogeneous, showed low standard deviations. In contrast, we have found a larger effect of the heterogeneity of the CF samples which results in a greater dispersion. Moreover, the standard deviation between aliquots did not follow a Gaussian distribution in case of CF samples, but exhibited a long tail for the most heterogeneous ones. For these, the vortexing procedure was probably too short or too gentle to reach a complete homogenisation. A complementary study would clarify the influence of the homogenisation parameters, such as vortex duration, on the dispersion of rheological results in natively heterogeneous samples. At the structural scale, microrheological investigations^15–17^ would provide insight on the intimate mechanisms involved during homogenisation, for instance to better understand how the inclusions interleave within the matrix and how it affects the mechanical properties of mucus locally.

Finally, we also characterised the temporal evolution of CF sputa. While purulent samples see their elastic and viscous moduli reduced by about half in the first 6 h, the semi-purulent samples remain unaffected. This contrasted result suggests that sputum degradation is mainly influenced by its biological load and not by its oxidation^32^. In particular, the presence of an immune response, evoked in this paper through the sample’s purulence, was already identified as a key contributor to static rheology^9^. The observed drops of $G'$ and $G''$ in CF and NCFB sputum coincide with the rheological degradation on acute asthma expectorations measured by Innes et al.^30^. Moreover, the degradation of the gel’s network was observed to break in the first 6 h after collection. To understand this stabilisation, comparing these data with the evolution of the neutrophil and bacteria population could be insightful. Practically, this result stresses the importance of quickly analysing sputum samples, and especially the purulent ones, before structural evolution occurs. The importance of purulence also scores the limitation of the current protocol to determine it by visual inspection as it is yet estimated subjectively. Defining purulence through a quantitative scale would allow a more rational classification.

Sputum rheology provides objective and quantitative properties, with relatively low variability in the measured quantities, provided that samples are properly vortexed immediately after collection. While access to a rheometer may be a constraint in some laboratories, the ability to freeze samples at $-80\,\,^{\circ }\hbox {C}$ without losing their rheological properties makes it possible to overcome this limitation. Several practical points should also be taken into consideration when performing rheological measurements. First, samples should be correctly loaded into the rheometer as incorrect placement is a major source of measurement artefact. Second, measurements should be carried out promptly as mucus samples are prone to dehydration at physiological temperature, which greatly increases the variability of rheological measurements^35^. The use of commercially available solvent traps attenuates this difficulty^35^. Third, high shears at the upper end of the amplitude sweep protocol may partly drive the sample out of the geometries, making the rheological results unexploitable in this limit. This is the reason why we limit our analysis to the critical point, when the sample starts flowing, without considering the flowing region itself. All considered, practical difficulties can be quite easily overcome, making sputum rheology a reliable method that could be broadly exploited in biophysical and preclinical research.

Overall, the present investigation draws guidelines towards a justified protocol for sputum rheometry. The existence of such protocol would benefit to both fundamental and clinical studies as the results given by different groups could be easily contrasted and related without concerns about the methodology. For instance, understanding the relation between healthy and diseased mucus composition with its structure and flowing abilities could help first to understand the influence of viscoelasticity in mucus clearance. Then, the study of sputum rheology could also help to assess which elements from its composition (bacterial load, immune response, DNA, mucins, etc.) are abnormal. In clinical context, the personalised surveillance and monitoring of muco-obstructive lung diseases is still a major challenge due to the lack of quantitative biomarkers, especially on the early detection of exacerbations^36^ and its immediate treatment. The demonstrated practical ability to perform unified rheological measurements could help tackling this challenge.

## Supplementary Information

Supplementary Information.

## Data Availability

The datasets used and/or analysed during the current study are available from the corresponding author on reasonable request.
