# Supplementary material for: Sputum handling for rheology
Source: Sci Rep. 2023 May 11;13:7695. doi: 10.1038/s41598-023-34043-9 (PMC10173912; doi:10.1038/s41598-023-34043-9)
Supplement: Supplementary file 1 — Supplementary Information. [file 41598_2023_34043_MOESM1_ESM.pdf]

Supplementary material to *Sputum handling for rheology*

Patients' data

We have included 36 expectorations collected from the "Centre de Ressource et de Compétence de Mucoviscidose" at the Grenoble Alpes University Hospital. 26 were obtained from adult CF patients and 10 from NCFB patients, who regularly come to the hospital for medical follow-up. Table S1 collects patients' data including gene mutations for the CF patients, demographic data as gender and age, body mass index (BMI), spirometry information: forced expiratory volume in one second (FEV1) and residual volume (RV) in percentage and expectoration's purulence by visual inspection as described by Murray *et al.*[1]

Patient	Genotype	Gender	Age	BMI (kg/m^2)	FEV1 (%)	RV (%)	Purulence
CF1	DF508, DF508	M	23	21	72	208	Purulent
CF2	DF508, DF508	F	28	24	73	202	Purulent
CF3	DF508, DF508	F	34	20	75	–	Purulent
CF4	DF508, R1128X	M	25	16	41	382	Semi-purulent
CF5	DF508, R334W	F	29	17	62	232	Purulent
CF6	DF508, indetermined	F	31	17	27	252	Semi-purulent
CF7	DF508, L732X	M	23	20	46	213	Semi-purulent
CF8	DF508, DF508	M	52	20	37	193	Purulent
CF9	DF508, DF508	F	42	19	47	263	Purulent
CF10	DF508, DF508	F	36	18	29	256	Purulent
CF11	1717-1G>A, DF508	M	30	24	79	197	Purulent
CF12	DF508, DF508	M	27	21	75	95	Purulent
CF13	DF508, DF508	F	26	22	58	267	Purulent
CF14	1717-1G>A, 3849G->A	M	34	23	107	144	Purulent
CF15	DF508, A455E	M	46	23	24	196	Purulent
CF16	DF508, R1128X	M	25	16	45	257	Semi-purulent
CF17	DF508, DF508	F	26	22	56	191	Purulent
CF18	2118del4, S902R	F	50	22	38	221	Purulent
CF19	DF508, V232D	M	52	23	99	85	Purulent
CF20	DF508, DF508	F	29	24	72	193	Semi-purulent
CF21	DF508, A455E	M	46	22	22	203	Purulent
CF22	DF508, 1811+1	M	19	18	62	190	Semi-purulent
CF23	DF508, DF508	M	28	25	129	155	Semi-purulent
CF24	DF508, DF508	M	31	20	94	17	Purulent
CF25	DF508, A455E	M	46	23	26	218	Purulent
CF26	DF508, DF508	F	27	22	80	143	Purulent
NCFB1		F	28	20	44	188	Purulent
NCFB2		F	37	20	44	85	Semi-purulent
NCFB3		F	19	25	86	113	Purulent
NCFB4		F	45	25	73	138	Purulent
NCFB5		F	37	25	58	124	Purulent
NCFB6		F	29	20	30	210	Purulent
NCFB7		F	45	24	75	132	Purulent
NCFB8		F	29	20	45	–	Semi-purulent
NCFB9		F	23	19	37	155	Semi-purulent
NCFB10		F	29	24	41	146	Purulent

Table S 1. Cystic Fibrosis (CF) and NCFB (NCFB) patients included in this study. The table gives data of patients' demographic, spirometry and purulence.

Vortex procedure

The collected expectorations were vortexed with Vortex Genie ™ 2 from Scientific Industries SI ™. The sample was vortexed gently increasing the stirring intensity until the sample visually takes the shape of a torus and starts flowing (see Figure S1). Therefore, the maximal speeds used were dependent on the sample but ranged from 1000 to 3000 rpm. This vortex intensity was then maintained during 30 s.

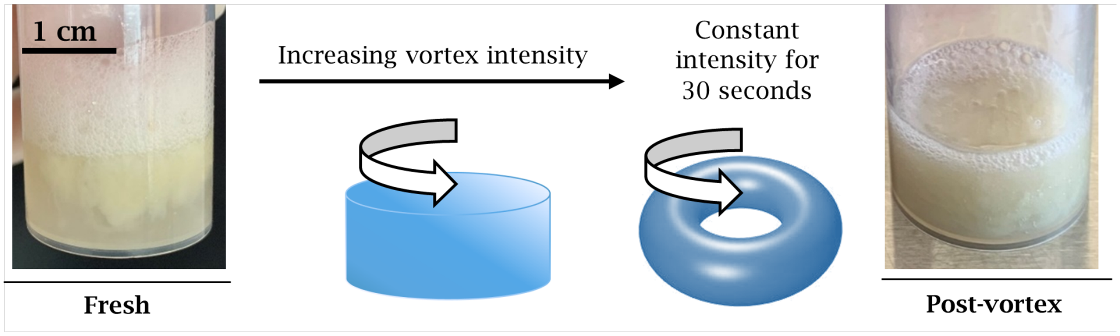

Figure S 1. CF sputum sample collected during a physiotherapist session in the CHU Grenoble Alpes before and after vortex. A schematic image shows the torus shape created when slowly increasing the vortex intensity. Once this torus is obtained, the intensity is kept constant during 30 seconds.

Expectorations

Figure S2 presents typical photographs of the fresh collected expectorations, ordered from lower to higher $g'_{5\%}$ standard deviations between replicates. The CF high variability samples do not seem visually more heterogeneous. Therefore, the vortex protocol should be optimised to reduce the high variability between replicas even when the differences in heterogeneity are not perceptible at sight.

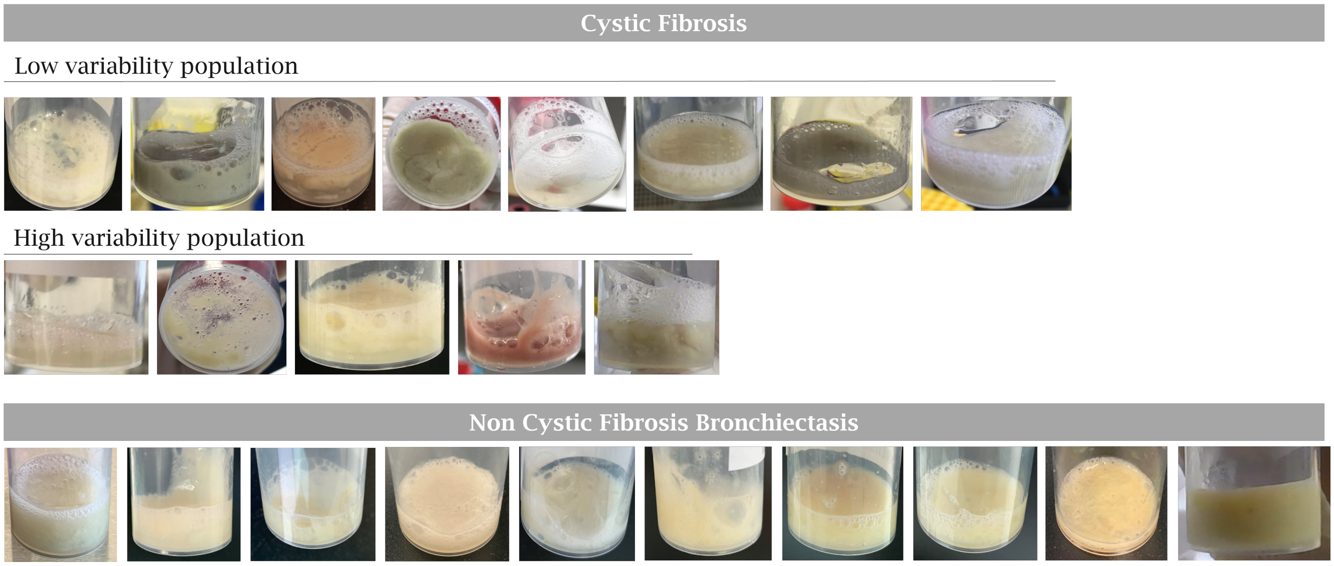

Figure S 2. Typical images of the fresh expectorations after collection, ordered by increasing dispersion in elastic modulus.

Treatment mean equality

We have carried out bilateral t-tests to study the mean equality between control (non-heated, non-frozen and vortexed) samples with treated samples. Heated samples have not been included as only one sample out of three showed a crossover, therefore d_c and s_c values are only available for this single sample.

Figure S3 displays whiskers plots for control (white) and treated samples (coloured). The p-values for the t-tests are indicated for each variable and condition. We can statistically accept no significant differences between the control and treated groups considering a confidence of 0.1. However, it is clear that, while the freezing methods show high p-values (> 0.75), the effect of not vortexing CF expectorations leads to values much closer to the acceptance level and therefore a relatively high risk of wrongly rejecting the alternative hypothesis (mean inequality).

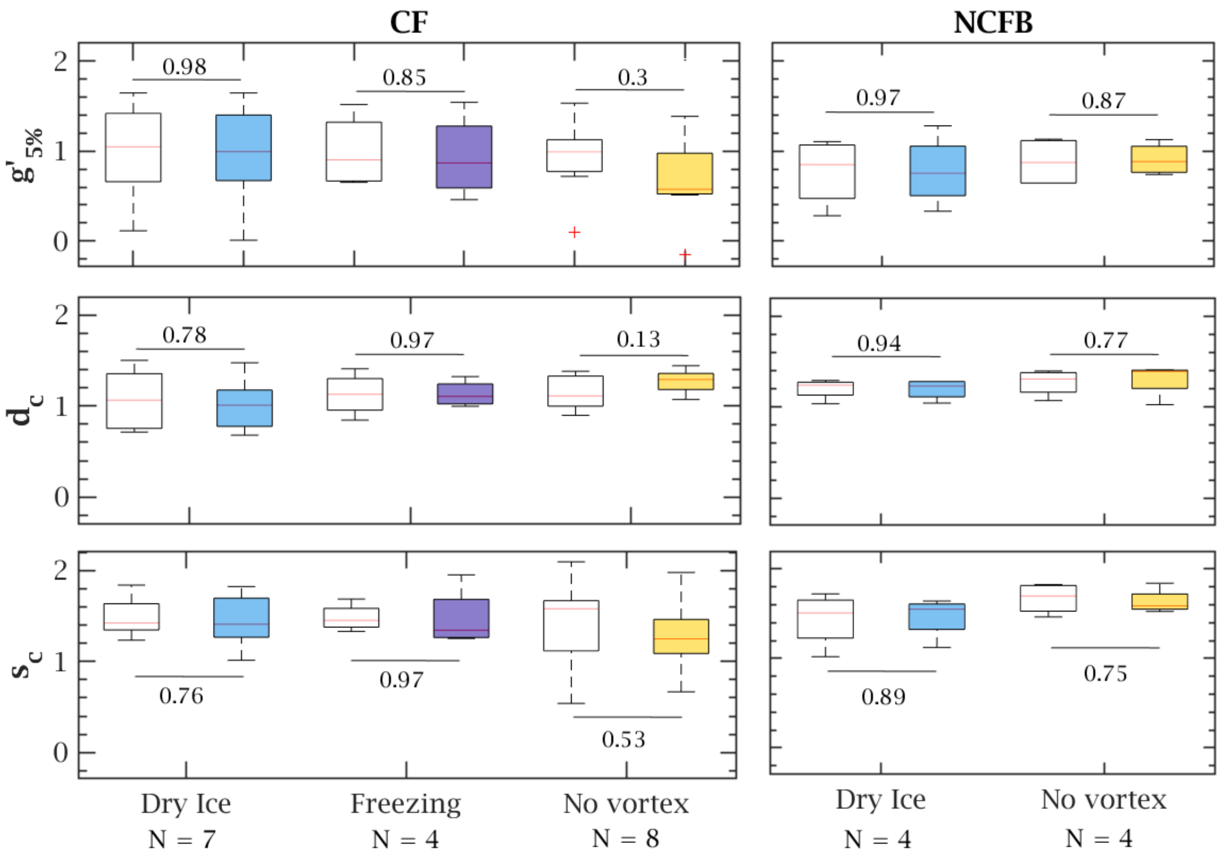

Figure S 3. Whiskers plots comparing control (white) and treated (coloured) samples. The central mark shows the median and the borders of the box designate the first and third quartile (25 and 75 % of the values). The obtained p-values for the mean equality bilateral t-tests are displayed for each condition.

References

1. Murray, M. P., Pentland, J. L., Turnbull, K., MacQuarrie, S. & Hill, A. T. Sputum colour: A useful clinical tool in non-cystic fibrosis bronchiectasis. *Eur. Respir. J.* **34**, 361–364, DOI: 10.1183/09031936.00163208 (2008).
